# Integration of ZnO nanorods with silver ions by a facile co-precipitation for antimicrobial, larvicidal, and ovicidal activity

**DOI:** 10.1186/s12896-023-00790-w

**Published:** 2023-07-20

**Authors:** Elsayed Elgazzar, H. A. Ayoub, Z. A. El-Wahab, Wageha A. Mostafa

**Affiliations:** 1 Department of Physics, Faculty of Science, Suez Canal University, Ismailia, Egypt; 2 Entomology Section, Zoology Department, Faculty of Science, Zagazig Universiry, Zagazig, 44519 Egypt

**Keywords:** Ag/ZnO NRs, Antimicrobial, Co-precipitation, Energy gap, Larvicidal, Ovicidal

## Abstract

**Background:**

Infectious diseases prompted by micro-organisms such as fungi, parasites, or microbes, have influenced many countries’ public health causing death. Scientists declared that metal oxide composites have various advantages in the medical field such as the antimicrobial feature has freshly been revealed as well as its role in suppressing mosquito population.

**Methods:**

In this work silver doped zinc oxide nanorods (Ag/ZnO NRs, 10 wt.%) were prepared by simple chemical route, and their microstructural characteristics were investigated by XRD, EDX, SEM, and TEM techniques. The antimicrobial, larvicidal, and ovicidal of the synthesized nanocomposites were examined.

**Results:**

The synthesized nanocomposite exhibited binary phase of crystallite size 112 nm was calculated from Williamson-Hall method. EDX spectrum revealed the purity of the composite consists of Zn, O, and Ag elements. The SEM and TEM micrographs showed the particles in nanorods with high density on the surface. The energy gap $({\mathrm{E}}_{\mathrm{g}})$ was evaluated from the UV–Vis absorbance in the range from 2.90 $-$ 3.08 eV inside the visible spectrum. The antimicrobial activity of the nanorods was examined against Gram-positive bacteria (*Staphylococcus aureus* and *Bacillus subtilis*) with inhibition zones 10.5 and 14.5 mm, respectively. Whereas gram-negative bacteria (*Escherichia coli, Salmonella Typhimurium,* and *Pseudomonas aeruginosa*) were 14 and 17 mm, respectively. Further, *Candida albicans* was investigated with inhibition zone 7.5 mm. Besides, the insecticidal impact of the nanocomposite against *Culex pipiens* larvae was performed at 30 mg/l causing 100% larval mortality with LC_50_ (11.78 mg/l). The micrograph images showed deformations in the larval body as well as egg resulting in zero egg hatchability.

**Conclusion:**

The findings approved that synthesized nanorods have a significant impact on controlling pathogens that impart different diseases to humans and the environment.

**Graphical Abstract:**

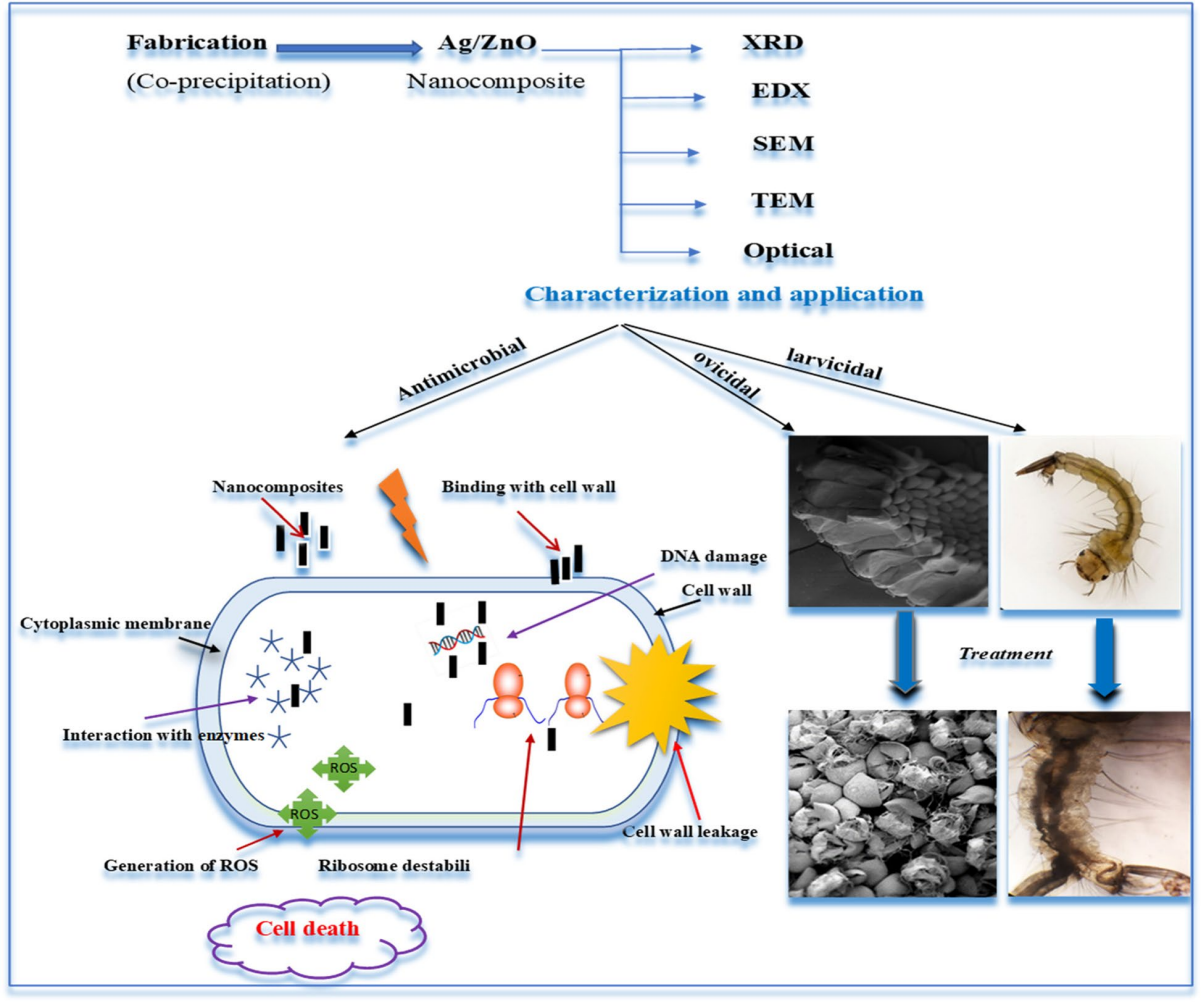

## Background

The excessive use of traditional energy sources led to an increase of pollution, global warming and consequently spread many infectious diseases. According to the international health organization report, about three million people are dying around the world due to microbial contamination including bacteria and fungi. Moreover, mosquitoes are the most prevalent type of vectors, they can transmit dangerous diseases posing harm to human health and environment. *Culex pipiens* (Diptera: Culicidae) is the lymphatic filarial vector which is the main reason for increasing number of individual mortalities. On the other side, the development of pathogenic microbial and vectors resistance to antibiotics and traditional synthetic insecticides results in infectious outbreaks which became difficult to control, particularly with recent climate changes [1–3]. Because of this great threat, the scientific community and health organizations worldwide are working on innovative novel strategies for suppressing the spread of diseases. Nanomaterials have been demonstrated as a new discipline with a significant impact on human life owing to their ability to withstand harsh process conditions and influential applications in medicine, biosensors, catalysis, and pharmaceuticals [3, 4].

Nanometal oxides like zinc oxide (ZnO), magnesium oxide (MgO), copper oxide (CuO), and silver oxide (Ag_2_O) have remarkable attention in biological field thanks to their eco-friendly toward humans, animals, and plants. ZnO nanoparticles of low toxicity, biocompatibility, chemical and thermal stability have been utilized as antimicrobial agents in medicine and food packaging for inhibiting microbial growth. Despite these advantages, ZnO nanopowder displayed some drawbacks including aggregation of the nanoparticles on the surface and wide optical band gap. Several literatures showed that incorporation of ZnO with Nobel dopants comprising Au, Sm, Ag, Ru, and Pd will improve its chemical and physical properties [5–7]. Because of the p-type nature of ZnO when doped with silver ions (Ag^+^). Doping ZnO with high Ag^+^ concentrations is beneficial in diffusion of majority electrons and holes as well as enhancement the chemical stability. Besides, the presence of Ag^+^ inside ZnO prevents electron–hole recombination and increases the free radicals making silver one of the best dopants. Furthermore, silver nanoparticles were applied in medicate for wound dressings, care of eye, anti-inflammatory and antimicrobial agent dependence of release large amounts of silver ions (Ag^+^) that easily interact with bacteria proteins causing death [6–9].

**Fig. 1:**
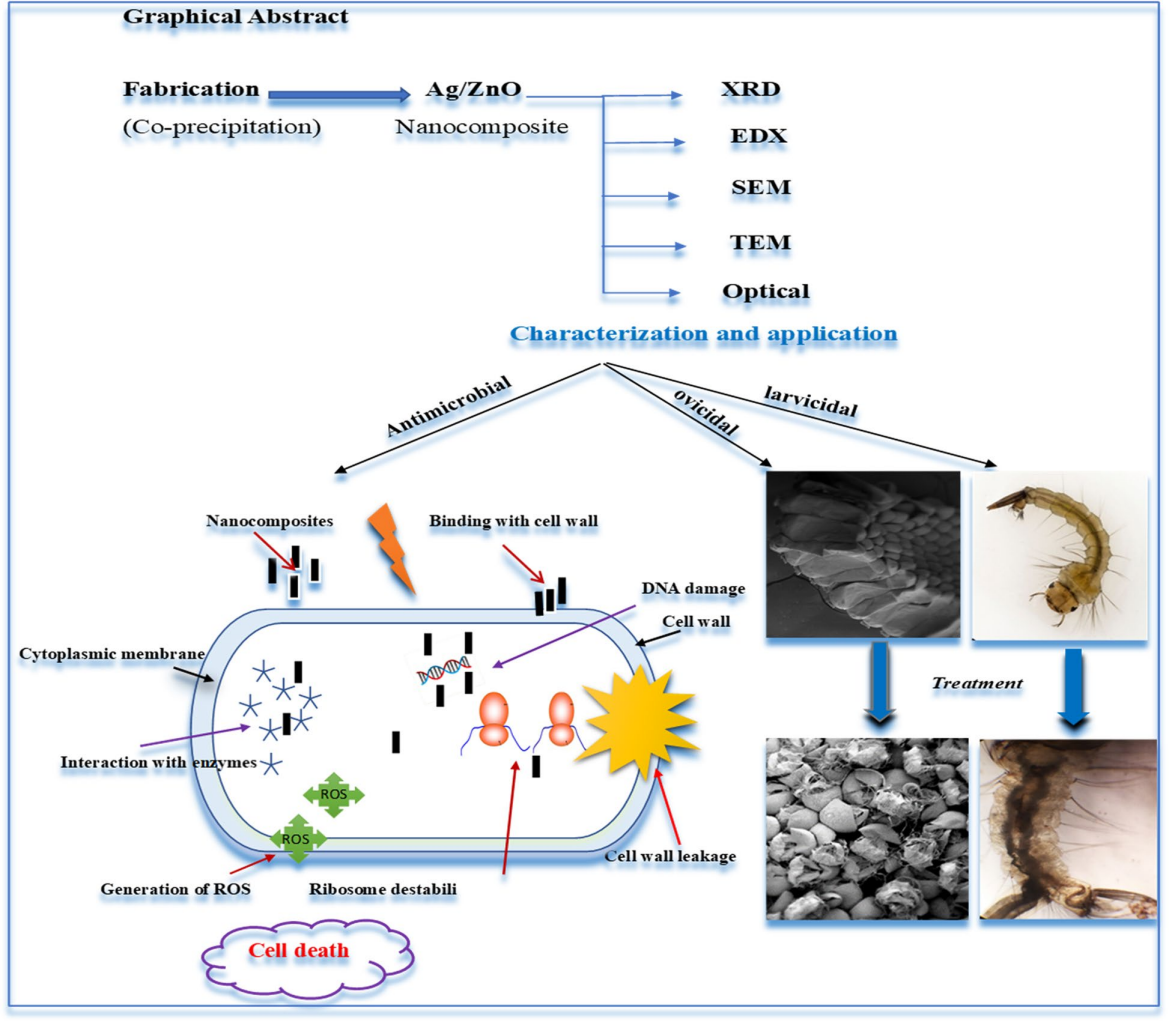
The scheme of impact the nanocomposite Ag/ZnO on bacteria, mosquito larvae, and egg

The nanocomposite Ag/ZnO exhibited a significant antibacterial capacity against pathogenic bacteria; *Escherichia coli*, *Pseudomonus aeruginosa*, and *Staphylococcus aureus.* Pavithra et al*.* (2022) have fabricated Ag/ZnO nanorods (NRs) with different concentrations via ultrasonic assisted co-precipitation approach for anticancer and antimicrobial activity [10]. In the present study, adding 10 wt.% Ag contents inside ZnO framework led to release more Ag^+^ and Zn^2+^ as well as production of reactive oxygen species (ROS) which have high toxic effect on bacterial cells and membranes [10, 11]. Numerous methods are utilized for producing nanocomposites such as chemical vapor deposition, spray pyrolysis, thermal decomposition, solvothermal method, precipitation, pulsed laser ablation, sol–gel, combustion method, microwave synthesis, hydrothermal treatment. Among them, the chemical co-precipitation approach demonstrated a great contribution to modern biotechnology owing to its friendly environment, modular and easy scaling up. It is easy to control shape, composition, purity, and particle size by monitoring annealing temperature, medium pH, and precursor ratio [11–13]. In the current investigation, structural-morphological and optical characteristics of ZnO were modulated by silver ions to support antimicrobial and larvicidal development. The impact of synthesized nanorods on microbes and mosquito larvae is illustrated in Fig. 1.

## Materials and Methods

### Chemicals and Reagents

Zinc acetate dihydrate (C_4_H_12_O_6_Zn, 98% extra pure), silver nitrate (AgNO_3_, min. 97%), Ammonium hydroxide solution (NH_4_OH, 25%), Cacodylate buffer, Glutaraldehyde, Osmium tetroxide (OsO_4_), Ethanol, and Acetone. Chemicals and reagents were purchased from Merck and Alfa Aesar company, Germany.

### Preparation of 10 wt.% Ag/ZnO NRs

Ag/ZnO was prepared by dissolving 2.15 g zinc acetate dihydrate (C_4_H_12_O_6_Zn) in 10 ml double distilled water using magnetic stirrer for 2 h without heating. In a dark room, 0.15 g silver nitrate (AgNO_3_) was dissolved in 10 ml double distilled water. Thereafter, the silver nitrate was gradually added to the zinc acetate solution with continuous stirring for 3 h at constant speed 800 rpm. Then, 15 ml ammonium hydroxide solution (NH_4_OH, 25%) was slowly added drop by drop to the metal salt mixture with continuous stirring at room temperature until a homogenous white gray solution was formed at pH $=$ 11. The resulting precipitate powder was filtered, washed many times using distilled water, then left to dry at 80 °C overnight. To obtain the nano-composite, the resulting powder was calcined at 400 °C in a muffle furnace for 2 h.

### Characterization of the synthesized nanocomposite

The crystal structure of Ag/ZnO was identified by the X-ray diffractometer (XRD, Rigaku Smart Lab.); operating at an accelerating voltage 35 kV, 25 mA with CuKα radiation (λ $=$ 1.540 Å). The chemical composition was checked using energy dispersive X-ray analysis (EDX; Helios Nanolab. 400). Morphological nature, distribution, and average particle size were visualized by scanning electron microscopy (SEM; Helios Nanolab. 400) and transmission electron microscopy (TEM; Hitachi-H-7500). The energy gap of the fabricated thin films was estimated from the UV–Vis absorbance spectra using spectrophotometer Jasco (V-570).

### Antimicrobial activity

Antibacterial activity of prepared sample was studied by disc diffusion method against selected microbes such as *Staphylococcus aureus*, and *Bacillus subtilis* (gram-positive), and *Escherichia coli*, *Salmonella typhimurium* and *Pseudomonas aeruginosa* (gram-negative) and *Candida albicans* (fungus). Whatman No.1 filter paper discs of 6 mm diameter were immersed with 30 μg Ag/ZnO NRs for each strain and left overnight after that the presence or absence of zones of inhibition around the disks was measured. The zones of inhibition were recorded after incubating the plates at 37 °C for 24 h [14]. Each test was carried out in triplicate and the data were subjected to statistical analysis as well as using Gentamycin (4 µg/ml) and Ketoconazole (100 µg/ml) as a positive control of bacteria and fungi, respectively.

### Mosquito rearing

For preliminary screening, Egg rafts of *C. pipiens* were brought from the Entomology institution, Cairo, Egypt. They are kept in plastic bowls filled with water for hatching. The larvae were fed on fish food and grown in plastic trays (30 × 25 x 5 cm). The colony was reared at a temperature of 26 ± 2 °C, relative humidity (RH) of 80 ± 5%, and photoperiod 14 L: 10 D. The adult mosquitoes were kept in cages (30 × 30 × 30 cm) and fed with a 10% glucose solution. The different developmental stages of mosquito eggs and larvae were used for the bioassays.

### Larvicidal and ovicidal bioassay

The toxicity test was performed by placing 25 mosquito larvae into 200 mL of sterilized double–distilled water with synthesized Ag/ZnO NRs to the designed concentrations (10, 15, 20, 25, and 30 mg/L) into a 250 mL beaker. Moreover, egg rafts were exposed to NRs at 30 mg/l. Distilled water was used as a control for each individual concentration. The number of dead larvae was counted after 48 h of exposure, and the mortality percentage was reported from the average of five replicates, and egg hatching was observed [15]. Further, the mortality rates were calculated using the formula.

1 $$\mathrm{Mortality }\ ({\%}) = (\mathrm{No}.\mathrm{\ of\ dead\ larvae}/\mathrm{\ total\ number\ of\ larvae\ introduced}) \times 100$$

### Optic Microscopy and SEM

After 48 h of treatment, the treated larvae were washed in 95% alcohol and examined using a light microscope (CXL Binocular compound microscope optic). The treated larvae and non-hatched egg rafts were prefixed for 2 h in 2.5 percent glutaraldehyde, then rinsed for 15 min in 0.1 M cacodylate buffer (pH 7.2), and then post-fixed for 1 h in 1% OsO_4_ in the same buffer. Specimens were washed in buffer, dehydrated in an ethanol series, and treated in an acetone solution. They were sputter-coated with gold and examined using SEM (Model, Helios Nano-lab. 400).

### Statistical analysis

All results were revealed as the mean ± standard deviation (SD) of the mean values. Statistically significant difference was calculated using Chi square followed by regression co-efficient to compare with control group. Probit transformation analysis was used to estimate the LC_50_ and LC_90_. A significance level of *P* ≤ 0.05 was statistically significant.

## Results

### Microstructural, composition, and morphological analysis

**Fig. 2:**
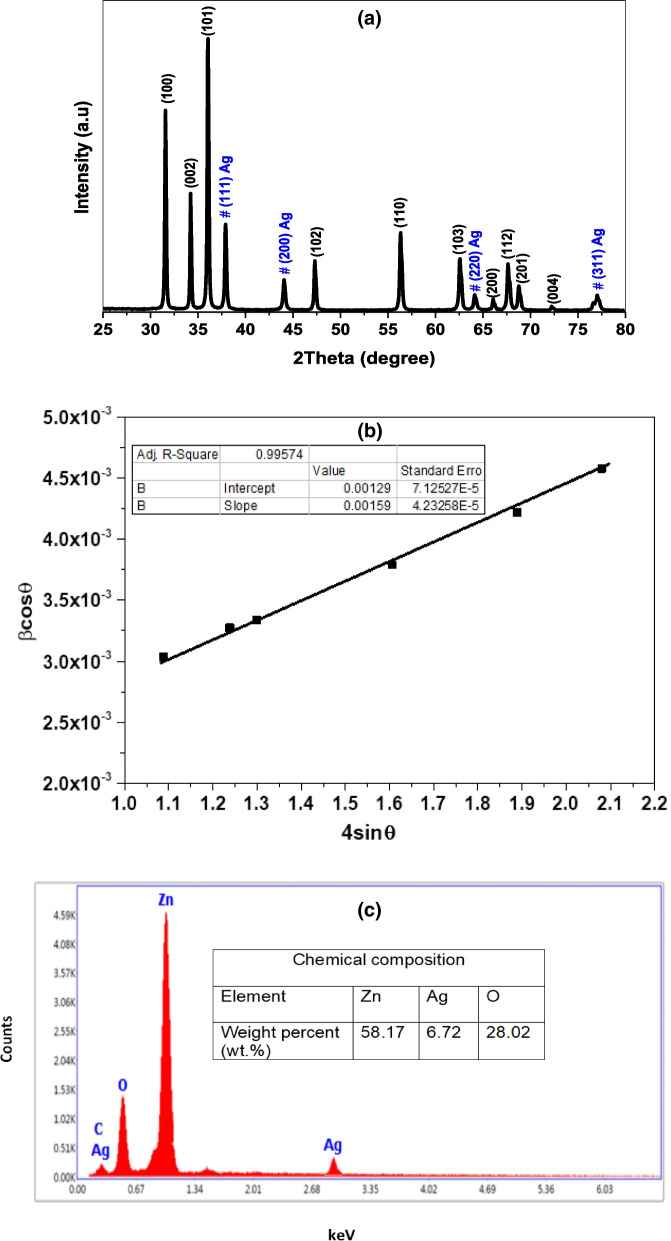
**A** XRD pattern, **B** W–H plot, and **C** EDX spectrum of the synthesized Ag/ZnO nanocomposite

The X-ray diffraction pattern was carried out in the $2\uptheta$ range of 25^o^ $-$ 80^o^ to investigate the crystal structure of Ag/ZnO. As observed in (Fig. 2A), the pattern displays sharp peaks with high intensity suggesting high crystallinity and a relatively big crystalline size. The binary phase confirmed the incorporation of silver ions inside the host ZnO lattice. The reflection peaks of the hexagonal wurtzite ZnO were detected at 2 $\uptheta =$ 31.56°, 34.21°, 36.01°, 47.25°, 56.29°, 62.63°, 66.08°, 67.63°, 68.82°, 72.27° corresponding to the planes 100, 002, 101, 102, 110, 103, 200, 112, 201, 004 respectively, according to JCPDS No. 36–1451, space group P63mc. In addition to four reflection peaks at 37.86°, 44.10°, 64.18°, 77.06° attributed to the face-centered cubic structure of silver nanoparticles, JCPDS file No. 04–0783. Despite the binary phase, no extra reflection peaks belong to impurities or unreacted ions are found in the XRD pattern. The crystallographic parameters like crystallite size $(\mathrm{D})$ and lattice strain $(\upvarepsilon )$ were evaluated from Williamson-Hall (W–H) using the following equation:

2 $${\upbeta\ \cos\uptheta }=\frac{\mathrm{k}\uplambda }{\mathrm{D}}+4{\varepsilon\ \sin\uptheta }$$

where, β is the full width at half maximum (FWHM) in radians, $\upvarepsilon$ is the lattice strain which measures the disturbance in nanostructure lattice due to doping concentration, $\uptheta$ is the Bragg’s scattering angle, λ is the wavelength of X-ray equal to 1.540 Å for CuKα radiation, and k is the crystallite shape factor $=0.94$. From W–H plot demonstrated in (Fig. 2B), the slope gives the strain whereas the intercept $=\frac{\mathrm{k}\lambda }{\mathrm{D}}$ provides the crystal size. Additionally, the dislocation density $(\updelta )$ and degree of crystallinity were defined by the relations:

3 $$\updelta =\frac{1}{{\mathrm{D}}^{2}}$$

4 $${\mathrm{X}}_{\mathrm{c}}=\frac{0.24}{\upbeta }$$

The values of $\mathrm{D},\upvarepsilon ,\upbeta ,{\mathrm{and X}}_{\mathrm{c}}$ of Ag/ZnO were summarized in Table 1. The EDX spectrum was performed to emphasize the elemental composition and purity of Ag/ZnO (Fig. 2C). The spectrum showed the main elements zinc (Zn), silver (Ag), and oxygen (O). The elements Zn, Ag, and O were found with weight percentage (wt.%) 58.17, 6.72, and 28. 02, respectively. The presence of carbon (7.09 wt.%) in the spectrum is attributed to the carbon coated grid.

**Table 1:** The XRD parameters of Ag/ZnO (10 wt.%) NRs

**Nanocomposite**	**D (nm)**	$\boldsymbol{\varepsilon} \times\textbf{10}^{-\textbf{4}}$	${\varvec{\updelta}}{\times} \textbf{10}^{\textbf{-5}}{\left(\textbf{nm}\right)}^{\textbf{-2}}$	${\textbf{X}}_{\textbf{c}}$
Ag/ZnO (10 wt.%)	112	16.00	8.00	65.00

**Fig. 3:**
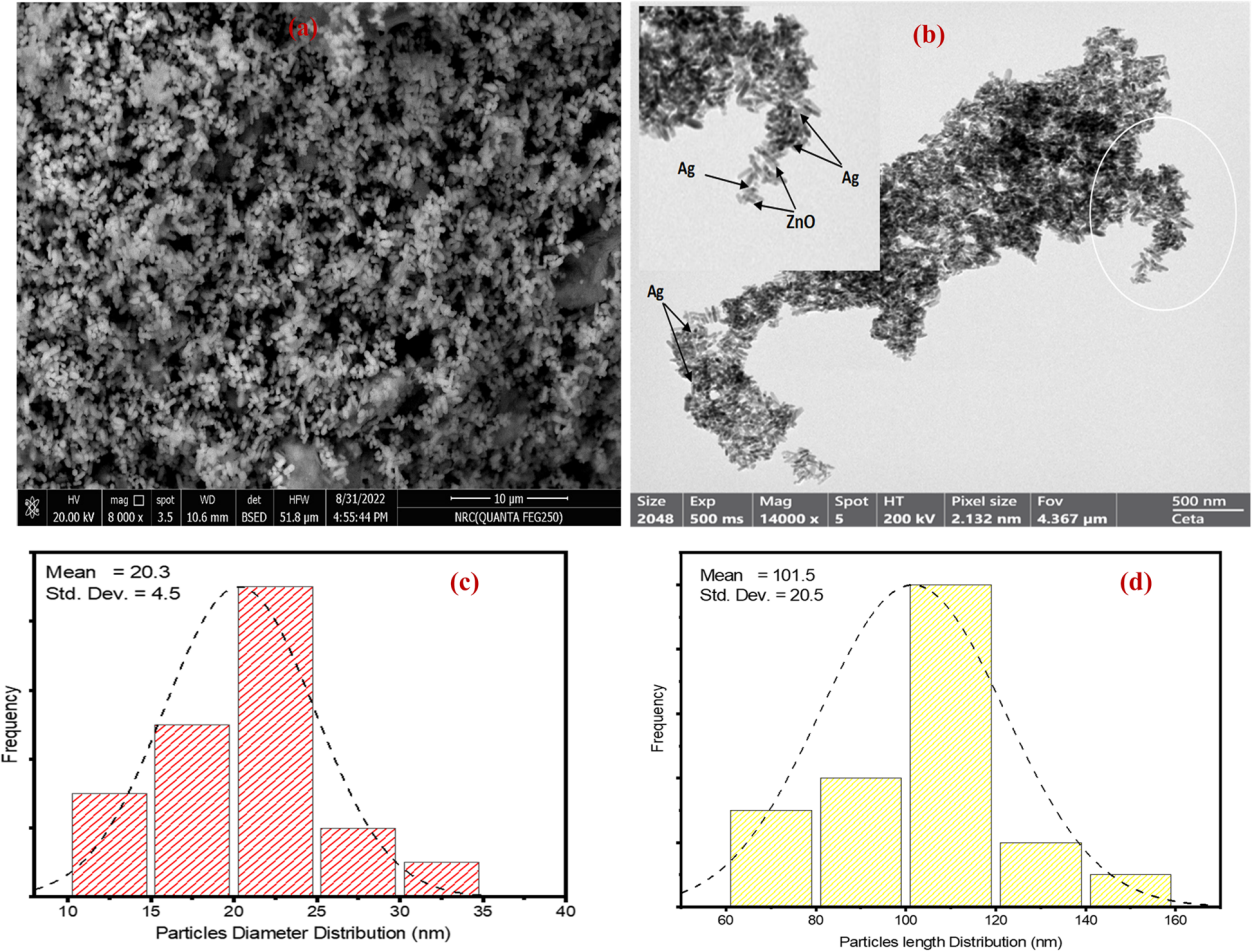
**A** SEM micrograph, **B** TEM micrograph, **C** Diameter and **d** Length distribution diagram of Ag/ZnO nanorods

Further, the surface morphology, particle distribution, and average length and diameter distribution diagram were identified by SEM and TEM micrographs. The SEM image shows the particles of the nanocomposite in tiny rods and high density (Fig. 3A). However, some of the nanoparticles have spherical shapes related to silver molecules. Moreover, the TEM image described the nanorods with large surface area, (Fig. 3B). Figure 3(C, D) illustrated the mean size of rod diameter and length distribution with aspect ratio equal to 5.

### Optical analysis

**Fig. 4:**
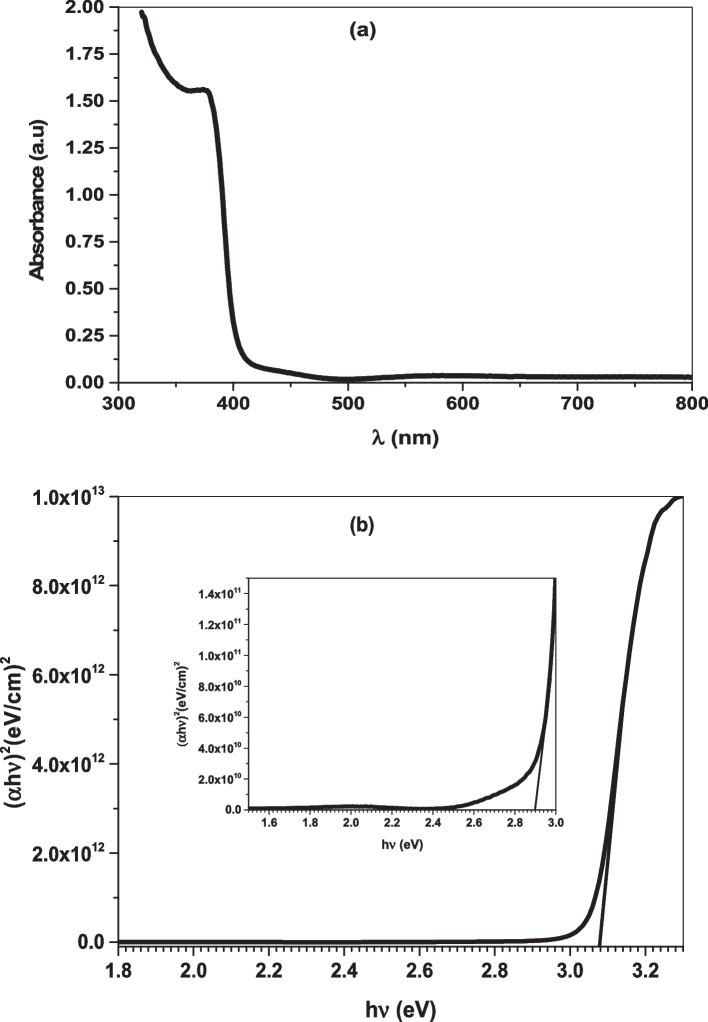
**A** Optical absorbance and **B** Energy gap (E_g) plot of Ag/ZnO thin film

The UV–Vis optical absorbance spectrum of Ag/ZnO thin film was recorded through the wavelength range from 350 $-$ 800 nm. The thin film has a sharp absorption edge at approximately 390 nm close to the visible region in which the absorption peak observed at 375 nm within the UV spectrum, (Fig. 4A). In order to define the type of electron transition of the semiconductor nanomaterials, the energy gap $\left({\mathrm{E}}_{\mathrm{g}}\right)$ was estimated from Tauc, Davis and Mott equation given by:

5 $$\left(\alpha \mathrm{h}\upsilon \right)=\mathrm{K}(\mathrm{h}\nu -{\mathrm{E}}_{\mathrm{g}})^{1/2}$$

By knowing the value of the absorption coefficient $({\alpha })$ from the relation:

6 $${\alpha }=\frac{2.303\mathrm{ A}}{\mathrm{t}}$$

where, t is the film thickness and A is the optical absorbance, h $\upnu$ is photon energy and K is a constant. From the ${(\alpha \mathrm{h}\nu )}^{2}$ against (h $\nu )$ plot in (Fig. 4B), the energy gap of Ag doped ZnO thin film was determined by extrapolating the linear part to the energy axis, the band gap was calculated to be 3.08 and 2.90 eV with direct allowed transition. The second energy gap may be owing to the localized defect levels that are generated below the conduction band inside the energy gap by silver ion dopants. Furthermore, the deposition of silver on the nanorods extends the range of the light absorption wavelength due to the localized surface plasmonic resonance effect.

### Antimicrobial activity

**Fig. 5:**
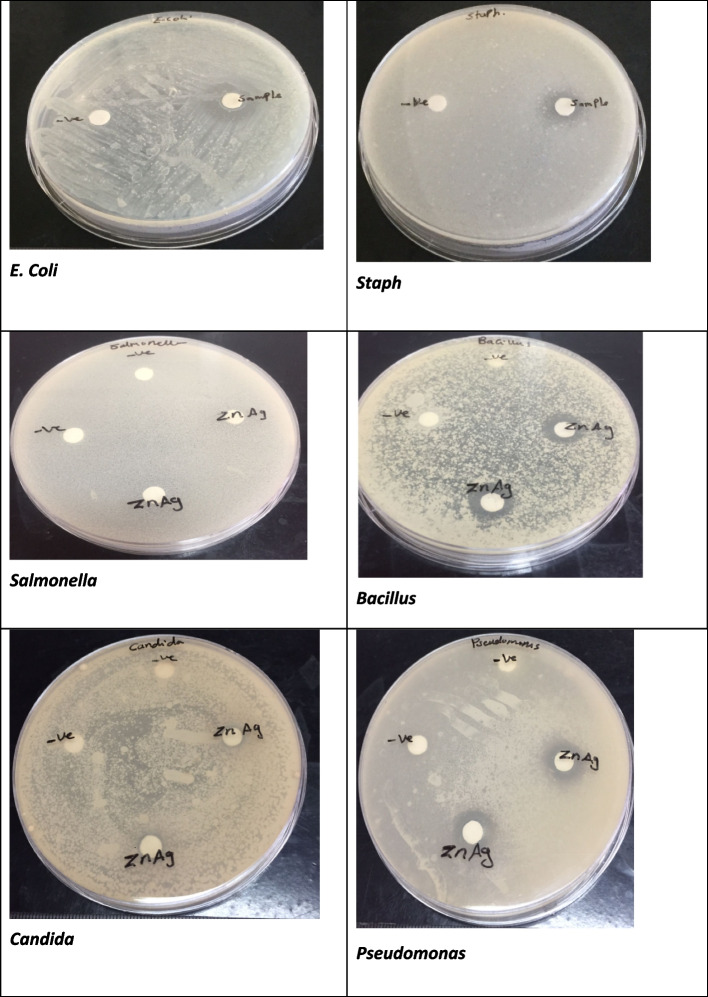
The inhibition zones indicating the antimicrobial activity of Ag/ZnO NRs against gram positive and gram-negative bacteria

Antimicrobial results revealed that the Ag doped zinc oxide nanoparticles are efficient against pathogenic microorganisms. As shown in (Fig. 5) and Table 2, *Pseudomonas aeruginosa* was the most sensitive among the tested microorganisms with 16 mm inhibition zone. Moreover, the inhibition zones of *Escherichia coli* ATCC 11229 and *Bacillus subtilis* were 14 and 14.5 mm, respectively*. Staphylococcus aureus* was less sensitive, with an inhibition zone of 10.5 mm, and the fungus, *Candida albicans* was 7.5 mm. However, the control drug exhibited higher activity compared to Ag/ZnO NPs. as demonstrated in the Table. 2*.* While *Salmonella typhimurium* showed resistance to the Ag doped zinc oxide nanoparticles. By biosorption, Ag/ZnO nanorods certainly enter through the bacterial cell membrane and bind with the negatively charged functional groups of the bacterial cell membrane (such as carboxyl and phosphate groups).

**Table 2:** Anti-bacterial activity of Ag/ZnO NRs against pathogenic bacteria and fungi (Disc Diffusion method)

**Inhibition zone diameter in mm**	**Ag/ZnO**	Tested microbial
Tested microbial	** (Mean ± SD) **	
**Gram positive bacteria**		**Gentamycin**
*Staphylococcus aureus*	10.5 ± 2.12	24 ± 1.07
*Bacillus subtilis*	14.5 ± 0.70	26 ± 0.65
**Gram negative bacteria**		
*Escherichia coli*	14 ± 1.41	30 ± 0.20
*Salmonella typhimurium*	Non sensitive	25 ± 1.09
*Pseudomonas aeruginosa*	16 ± 1.41	25 ± 0.86
**Pathogenic fungus Ketoconazole**		
*Candida albicans*	7.5 ± 0.70	20 ± 0.31
**Negative Control**	Non sensitive	

From the comparison, Table 3 illustrates that the prepared Ag/ZnO nanocomposites exhibited a good zone of inhibition in most microbes except *Bacillus subtilis* compared to metal oxide nanoparticles (ZnO Nps) and metal nanoparticles (Ag Nps).

**Table 3:** Tested microbial for the present work and the previous studies

**Tested microbes**	Ag/ZnO	ZnO Nps	Ag Nps
	present work	previous studies	
**Gram positive bacteria**			
*Staphylococcus aureus*	10.5±2.12mm	4 ± 0.82 mm (16)	8 ± 0 mm (17)
*Bacillus subtilis*	14.5 ± 0.70 mm	25 ± 0 mm (18)	25 ± 0 mm (18)
**Gram negative bacteria**			
*Escherichia coli*	14±1.41mm	0 (16)	15 ± 0 mm (19)
*Salmonella typhimurium*	Non sensitive	0	
*Pseudomonas aeruginosa*	16± 1.41m	0 (20)	0 mm (20)
**Pathogenic fungus**			
*Candida albicans*	7.5 ± 0.70 mm	0 (20)	0 mm (20)
**Control**	Non sensitive	0 (20)	0 mm (20)

### Larvicidal and ovicidal impact of nanoparticles against *C. pipiens*

**Fig. 6:**
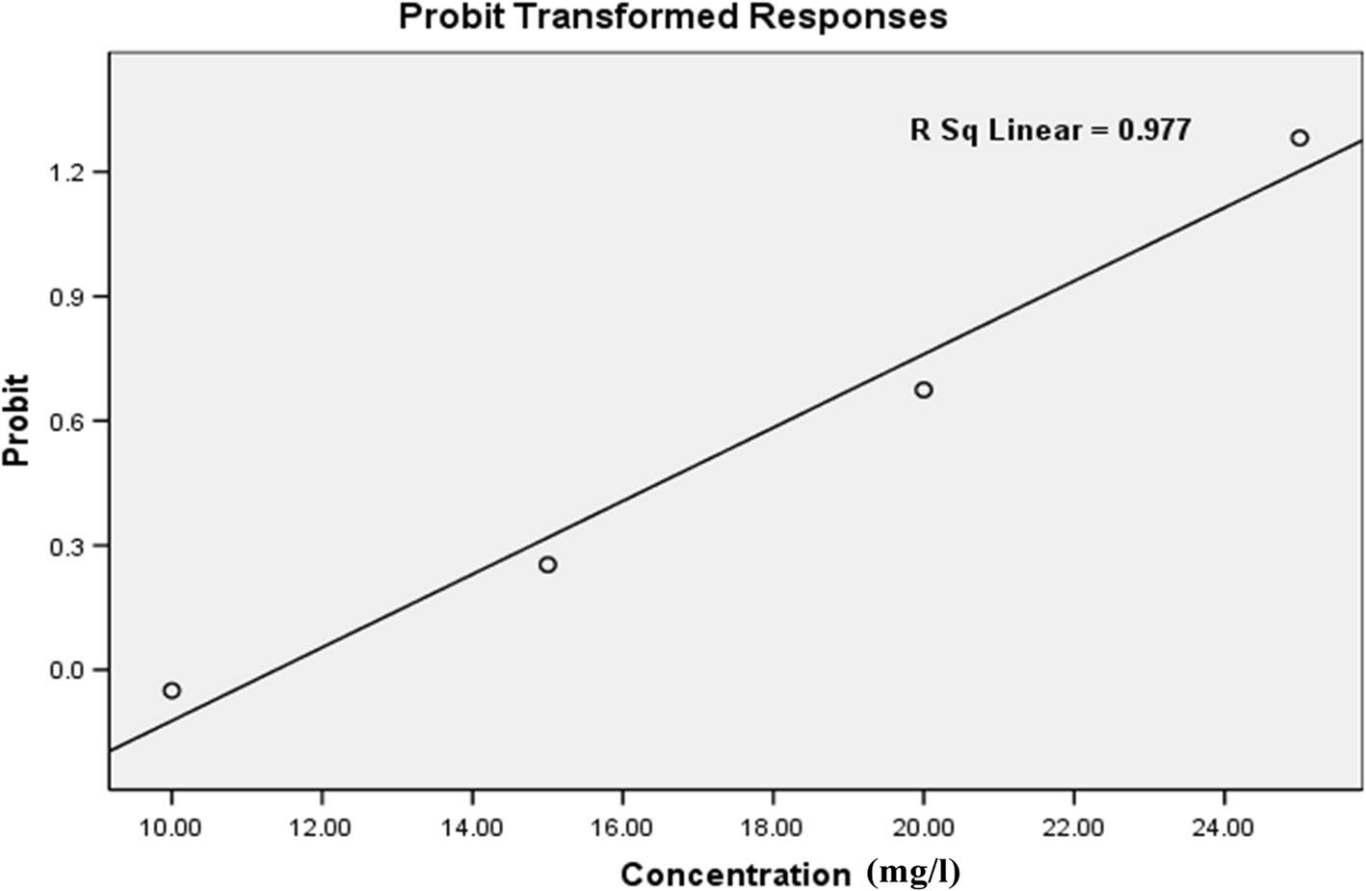
Probability analysis of mortality of C. pipiens mosquito larvae by Ag/ZnO NRs

The toxic effect of Ag/ZnO nanorods was examined on *C. pipiens* larvae. The third larval instars of *C. pipiens* have been subjected to the synthesized nanoparticles and the death rate was evaluated by little amounts of the nanoparticles (10. 15, 20, 25, and 30 mg/l). The doped Ag/ZnO NRs showed mortality rates from 48 ± 0.57 to 100% according to the concentration, Table 4. The mortality probability of the nanopowder on *C. pipiens* mosquito larvae were distributed in (Fig. 6). It was observed that larvicidal activity has been achieved by applying a small dose of nanomaterials. Moreover, LC_50_ and LC_90_ of Ag/ZnO against the larvae (*C. pipiens*) were 11.877 and 24.314 mg/l, respectively as presented in Table 4. Besides that, when eggs exposed to nanoparticles at the highest concentration showed zero hatchability. Control larvae observed no mortality, and eggs without treatment hatched normally. As summarized in Table 4, the insecticidal impact of the nanostructure against the larvae is dependent on the concentrations. Additionally, the regression equation suggested that there was a linear relationship between the mortality percentages and concentrations of Ag-doped ZnO nanoparticles.

**Table 4:** Dose-dependent larvicidal activity of Ag/ZnO NRs against *C. pipiens* species

NRs	Conc.	% Mortality	LC_50_ (LC_90_)	95% confidence limit		Regression	X^2^
	mg/l	mean ± SD	mg/l	LC_50_ (LCL-UCL)	LC_90_ (LCL-UCL)	Equation	(df = 4)
**Ag/ZnO**	10	48 ± 0.57					
	15	60 ± 0.40					
	20	75 ± 0.25	11.78 (24.31)	(4.98 -20.76)	(15.01 -32.67)	Y = -1.26 + X × 0.106	6.412
	25	90 ± 0.28					
	30	100 ± 0.00					

LC_50_ LC_90_: lethal concentration that kills 50 and 90% of the exposed *Cx*. *pipiens* larvae; UCL: upper confidence limit; LCL: lower confidence limit; X^2^ = Chi-square, *P* < 0.05, significant level, Mean value of five replicates, (SE) standard error. Control (distilled water), nil mortality

### Histopathological investigation of treated larvae and eggs

**Fig. 7:**
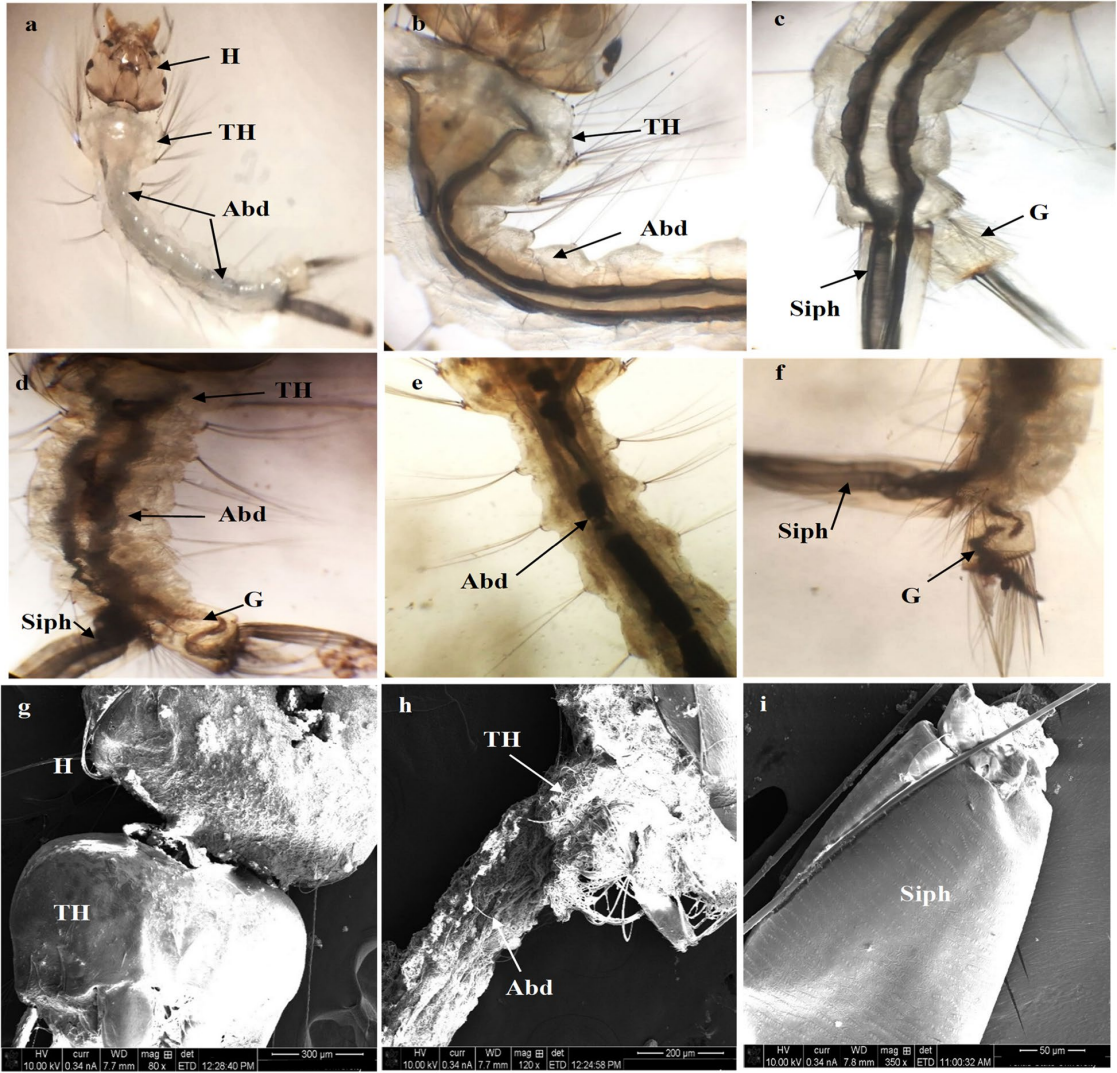
Light microscope photographs of the 3rd larval instars of Cx. pipiens. **A** normal larval body parts, **B** normal thoracic and abdomen with alimentary canal, **C** normal respiratory siphon and gills, **D** treated larvae with Ag/ZnO, **E** accumulate of nanoparticles in larval gut, **F** block of respiratory opening with nanoparticles (10 × 150). SEM images of treated larvae, **G** the deformation of head region, **H** malformed thoracic and abdomen parts with NPs, **I** blocking siphon with NPs. (Abd: abdominal segments, G: gill, H: head, Siph: siphon, TH: thoracic)

The potential larvicidal activity of the fabricated Ag/ZnO NRs was clearly observed under a light microscope and scanning electron microscope (SEM). The damage of the larval tissues is inferred by histopathology analysis after a 48-h exposure time at 30 mg/l, as observed in Fig. 7. Light microscope images of treated larvae revealed ruptures in the body, and it turned black and shrunk. Moreover, the accumulation of Ag/ZnO NRs was found in the entire alimentary canal of the larvae. Also, the nanoparticles blocked the respiratory opening as in (Fig. 7D, E, F). On the other side, the control larvae showed normal morphological features in body parts (Fig. 7A, B, C). Additionally, SEM images illustrate the impact of Ag/ZnO NRs on the larval body parts. This impact was observed as the deformation of head region (Fig. 7G), demolition of the abdominal region, deposition of nanoparticles on their integument (Fig. 7H). Accumulation of NRs in the siphon and gills which are the respiratory parts of the *Culex* larvae lead to suffocation and causes fatality to the larvae as seen in Fig. 7(I). Mosquitoes of the *Culex* species lay their eggs in the form of egg rafts that float in stagnant water.

SEM micrographs of control eggs show egg color is dark, elongate, and conical in shape, and slightly curved in their long axis with the tapered posterior end (P) exposed to the atmosphere and round anterior end (A) in direct contact with the water surface.

**Fig. 8:**
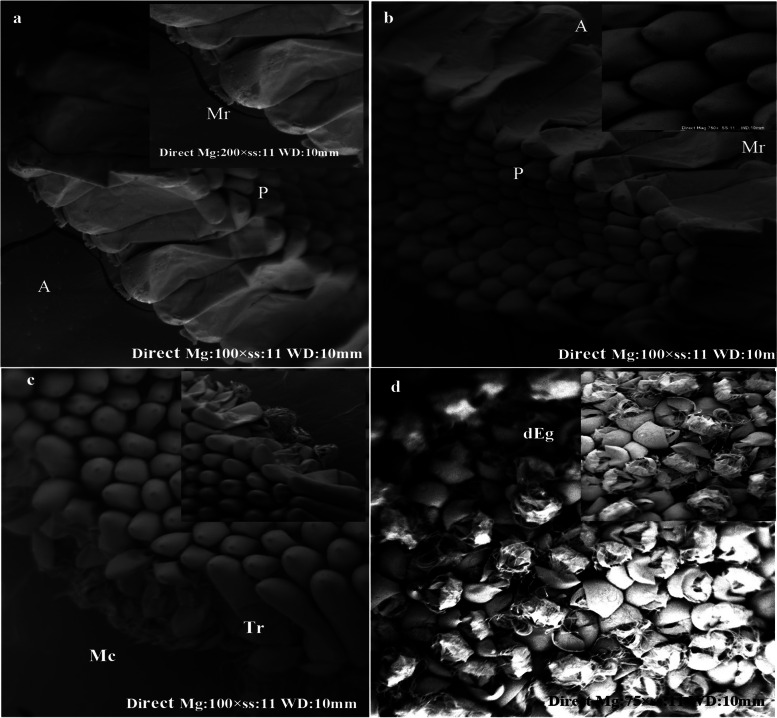
SEM micrographs of the control egg rafts mosquito Cx. pipiens **A, B** normal egg with cap-like circular micropylar and conical-shaped and **C, D** showed treated non hatched egg raft with malformed structure. (dEg: deformed egg, Mc: micropylar corolla, Tr: tubercular row)

Morphologically, the regular egg can be divided into cap-like circular micropylar and conical-shaped regions (Fig. 8A). In the anterior end, the micropylar apparatus is arranged in a flower-like shape which is considered the primary fertilization site and helps embryo survival (Fig. 8B). Figure 8(C,D) illustrated the effect of Ag/ZnO NRs on egg rafts, morphological changes in the eggshell, especially at the micropylar corollas, resulting in damage to the egg membrane and preventing egg hatchability.

## Discussion

It is well known that many factors impact on the nanostructured materials leading to the phase segregation such as calcination temperature, the difference in electronegativity, and the dopant ions ratio. Here, the large amount of Ag^+^ concentration that loaded inside the host ZnO framework as well as the large ionic radius of Ag^+^ (0.126 nm) compared to Zn^2+^ (0.074 nm) result in occupying the interstitial sites causing phase separation [16, 17]. The synthesized nanocomposite revealed a good crystalline nature of big size attributed to the rod like structure or the hexagonal shape of the nanoparticles. The dislocation density of the prepared composite was relatively large influenced by the high concentration of Ag ions [18–20]. The purity and element composition of Ag/ZnO were confirmed by EDX spectrum revealing that silver ions were successfully incorporated into ZnO sites. However, the peak observed at 0.277 eV is attributed to carbon coated grid [20, 21]. The topological features were investigated from SEM and TEM images showing that the surface nature in nanorods. These rods are tiny sharp, not uniformly distributed and have average length of approximately 101.5 $0\mathrm{ nm},$ diameter $20.30\mathrm{ nm}$ and aspect ratio $=$ 5 [22, 23]. The special dimensions and morphological properties of the nanorods enable them to be used as antimicrobial and anticancer agent with high performance [24, 25]. The optical study revealed that the nanocomposite of semiconductor nature in which the energy gap lies in the range of approximately 3 eV. The red shift in the optical band is attributed to absorption of the incident light energy and transfer of more electrons from the valence band to the conduction band generating free electrons into the lattice [26–28]. The increase of free charge carriers including reactive oxygen species (ROS) is so important in microbial and larvicidal activity [28, 29].

**Fig. 9:**
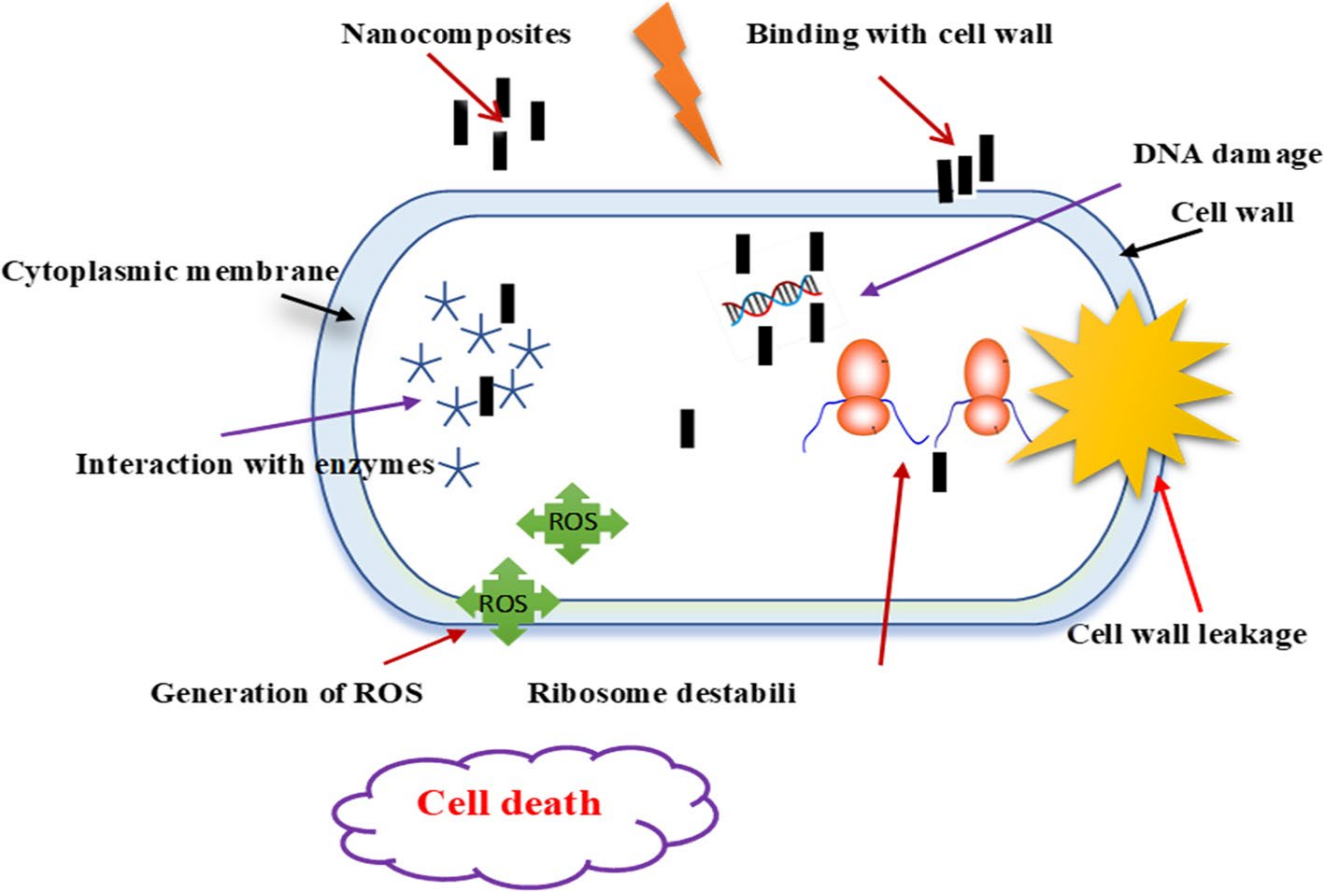
Antibacterial activity mechanism of Ag/ZnO NRs

In the present work, the antimicrobial activity of the Ag doped zinc oxide nanoparticles is efficacious against pathogenic microorganisms. These findings agree with earlier studies that demonstrated the antibacterial potential of zinc oxide nanostructure against pathogenic gram positive and gram-negative bacteria such as *Escherichia coli*, *Staphylococcus aureus*, *Pseudomonas aeruginosa*, and *Bacillus subtilis* [30–32]. Silver substituted ZnO nanorods are investigated for antibacterial ability in controlling *E coli* and *Staphylococcus aureus* growth [33]. Furthermore, the mechanism of action of Ag/ZnO nanocomposites involves the penetration and leakage of the bacterial membranes resulting in the release of reactive oxygen species (ROS) involving superoxide and hydroxyl radicals. This is due to the electrostatic attraction as well as their affinity to sulphur-containing proteins in the bacteria. Consequently, the nanoparticle ions are able to adhere to the cell wall and cytoplasmic membrane of the bacteria [34, 35]. Inside the bacteria ROS and nanocomposites are directed to DNA damage, lipid peroxidation, and protein oxidation can destroy bacteria [36, 37]. These intracellular functional disorders are initiated by the oxidative stress manipulated by ROS leading to cell death as illustrated in Fig. 9. Limited research investigations express the mechanism of electrostatic attraction [37, 38]. These results coincided with Olejnik et al. (2020) who reported that the ZnO NRs recorded higher toxicity as compared with spherical particles underlining the significance of particle size and shape for cytotoxicity [39]. Also, Yang et al. (2009) have shown that zinc oxide rods have the ability to penetrate through bacteria more easily than spheres [40].

The insecticidal effect of Ag/ZnO on *C. pipiens* larvae caused a significantly high rate of larval mortality in short periods of time. These results indicated that the nanocomposite can be developed as mosquito larvicidal agent, since the LC_50_ values were usually less than 15 ppm, while the nanoparticles prepared with plant extracts showed LC_50_ higher than 20–25 ppm [41, 42]. As depicted, a positive relationship between the higher concentration of nanorods and the larval body's absorption of a high quantity of nanoparticles which ultimately caused their death [43]. Owing to the aquatic habitation of *C. pipiens* larvae and egg-laying make all the body surfaces are exposed to a large amount of nanostructure suspension [44] consequently making them suitable stages to control mosquito vectors. Moreover, Ag^+^ in the intracellular spaces binds to sulphur containing proteins or to phosphorus-containing compounds like DNA, leading to the denaturation of some organelles and enzymes [45]. LC_50_ of ZnO rod shape against *Ae. aegypti* was ranged from 3.44 (1^st^ larva) to 14.63 ppm (pupa) with high larvicidal efficiency. Further, nanostructured zinc oxide exhibits 100% mortality of mosquito (larva IV) for (*Anopheles stephensi*), and (*Culex quinquefasciatus*) at 8 and 10 μg/ml [46]. The larvicidal activity of synthesized Ag NPs against *C. quinquefasciatus* larvae LC_50_ and LC_90_ were (14.70 and 28.96 ppm), respectively [47]. Literature reported that ZnO, CuO, MgO, AgO, and CeO_2_ nanoparticles have efficacy against mosquito species [48]. In the previous study, the zinc oxide nanostructure showed ovicidal, larvicidal activity against mosquito life stages through the synergistic influence of both zinc and oxygen ions. Zinc ions accumulate in mosquito life stages and once the amount of zinc is above the tolerance level, the metal accumulation leads to toxicity and inhibits their growth [49, 50].

The aggregation of Ag/ZnO NRs was illustrated in the entire alimentary canal of the larvae, and Ag/ZnO NRs are deposited in the respiratory parts of the *Culex* larvae which lead to larval death. A similar observation has been reported using cadmium, MnO_2_ and zinc oxide nanostructure against mosquito larvae [51, 52]. The present results showed that tissues suffer significant damage involving accumulation of the nanostructure in the thorax, abdomen, and gills as shown in Fig. 6 that are coincided with the literature [53] who reported that CuO NPs possess larvicidal activity on *C. pipiens* resulting in deterioration of the larvae's digestive canal, siphon, and gills. Additionally, the micropylar apparatus is arranged in a flower-like shape which represents the main fertilization site and helps embryo survival [54, 55]. The morphological deformation in the eggshell, particularly at the micropylar corollas, causing damage chorion and preventing egg hatchability [56]. Also, Ag/ZnO nanocomposite was more efficient as antimicrobial compared to metal and metal oxides [57–61].

## Conclusions

The Ag/ZnO composite was successfully synthesized by a scalable co-precipitation approach. The structural-morphological characteristics showed the particles in nanorods possess large surface area and aspect ratio $=5$. The optical analysis revealed that the thin film has a strong optical absorption within the visible region and an energy gap ~ 3 eV in the range of semiconductor nanomaterials. These exceptional physico-chemical features enabled the nanorods to be used as a potent agent against pathogenic bacteria and fungi as well as combating mosquito immature stages. The findings approved the high efficacy of the nanocomposite to control infectious diseases.

## Data Availability

The data that support the findings of this study are available from the corresponding author on reasonable request.
